# The Impact of Bariatric Surgery on Gut Microbiota Composition and Diversity: A Longitudinal Analysis Using 16S rRNA Sequencing

**DOI:** 10.3390/ijms26167933

**Published:** 2025-08-17

**Authors:** Radu Petru Soroceanu, Daniel Vasile Timofte, Sergiu Timofeiov, Vlad Ionut Vlasceanu, Madalina Maxim, Ancuta Andreea Miler, Andi Gabriel Iordache, Roxana Moscalu, Mihaela Moscalu, Irina Cezara Văcărean-Trandafir, Roxana-Maria Amărandi, Iuliu Cristian Ivanov, Alin Constantin Pînzariu

**Affiliations:** 1 Department of Surgery, “Grigore T. Popa” University of Medicine and Pharmacy, 700115 Iași, Romania; petru.soroceanu@umfiasi.ro (R.P.S.); daniel.timofte@umfiasi.ro (D.V.T.); madalina.maxim@umfiasi.ro (M.M.); ancuta-andreea_a_miler@d.umfiasi.ro (A.A.M.); andi.iordache@umfiasi.ro (A.G.I.); 2 Department of Surgery, “Sf. Spiridon” County Clinical Emergency Hospital, 700111 Iași, Romania; alin.pinzariu@umfiasi.ro; 3 Division of Cell Matrix Biology & Regenerative Medicine, School of Biological Sciences, Faculty of Biology, Medicine and Health, The University of Manchester, Manchester M13 9PL, UK; roxana.moscalu@postgrad.manchester.ac.uk; 4 Department of Preventive Medicine and Interdisciplinarity, “Grigore T. Popa” University of Medicine and Pharmacy, 700115 Iasi, Romania; moscalu.mihaela@gmail.com; 5 TRANSCEND Research Center for Fundamental and Experimental Development in Translational Medicine, Regional Institute of Oncology, 700483 Iaşi, Romania; trandafirina.bi@gmail.com (I.C.V.-T.); rpomohaci@iroiasi.ro (R.-M.A.); iuliuic@gmail.com (I.C.I.); 6 Department of Morpho-Functional Sciences II, “Grigore T. Popa” University of Medicine and Pharmacy, 700115 Iași, Romania

**Keywords:** RYGB, LSG, bariatric surgery, obesity, gut microbiota, 16S rRNA, microbial diversity, metabolic health

## Abstract

Bariatric surgery is considered the most effective treatment for obesity and its associated metabolic disorders, yet the underlying mechanisms are only partially understood. Evidence suggests that the gut microbiota plays an important role in metabolic regulation and can be significantly altered by bariatric and metabolic procedures. This prospective, single-center study aimed to evaluate the dynamic changes in the gut microbiota composition and diversity in obese patients undergoing two types of bariatric surgery: laparoscopic sleeve gastrectomy (LSG) and Roux-en-Y gastric bypass (RYGB). Fecal samples were collected at three time points—before surgery (T0), and at 3 (T3) and 6 months (T6) postoperatively—and analyzed using 16S rRNA gene sequencing targeting the V3–V4 regions with Illumina technology. Significant shifts in microbial diversity and structure were observed over time, indicating a trend toward microbiota normalization post-surgery. Notable changes included a reduction in the *Firmicutes*/*Bacteroidetes* ratio and increased relative abundance of *Actinobacteria*, *Proteobacteria*, and *Verrucomicrobia*. These alterations occurred in parallel with improvements in body mass index (BMI) and metabolic parameters. Our findings suggest that bariatric surgery induces favorable and sustained modifications in the gut microbiota, which may contribute to its therapeutic effects in obesity management.

## 1. Introduction

The human gastrointestinal tract hosts a vast and dynamic community of microorganisms, collectively known as the gut microbiota. It plays an important role in metabolic, immunological, and structural functions of the host [1]. This microbial ecosystem is increasingly recognized as a key factor in health and disease, particularly in metabolic disorders such as obesity [2]. Recent research has revealed that alterations in the gut microbiota composition—often referred to as dysbiosis—are associated with increased energy harvest, low-grade inflammation, and insulin resistance, all of which contribute to the pathogenesis of obesity [3,4].

Microbiome research involves the analysis of DNA from a biological sample. It is an essential approach in understanding the complex community of microorganisms present in a specific environment [5]. The main advantages are represented by the fact that it is not necessary to isolate and grow these microorganisms for their identification. Currently, such analyses are largely performed using next-generation sequencing (NGS) methods [6,7]. The bacterial 16S gene refers to a specific region of the 16S ribosomal RNA (rRNA) gene conserved in the bacterial genome [8]. This category is a molecular marker used to identify and classify bacteria and is part of the prokaryotic ribosome, playing a crucial role in the translation process [9].

When performing 16S sequencing, one or more of the nine variable (V) regions of the gene are targeted for analysis. Typically, in the absence of long-read sequencing technologies (PacBio or Oxford Nanopore), many researchers choose to amplify between one and three of these variable regions for sequencing using Illumina technology, which currently does not support complete coverage of the entire 16S rRNA gene. The selection of variable regions and primer sets significantly influences the results, as certain species may appear similar in certain regions and cannot be reliably differentiated [6,9].

Studies often choose primer sets that cover the V1–V3, V4–V5, or just the V4 region. However, the V3–V4 region is most commonly used, in part due to the higher degree of variability of these two regions combined compared to other regions, which improves species discrimination [9]. These two regions were also targeted in the present study. In addition, this preference is due to the ease of distinguishing between the various microorganisms present in the human gut. By comparing the obtained sequences with known sequences and classifying them in international databases, the bacteria present in the sample can be identified and classified [6].

Bariatric and metabolic surgical procedures (BMSs) are considered by many a safe and effective treatment option for severe obesity and its associated comorbidities [10,11,12]. Growing evidence suggests that beyond mechanical restriction and malabsorption, bariatric procedures may exert beneficial effects through modulation of the gut microbiota [13,14]. However, the direction and significance of these microbiota shifts remain incompletely understood and sometimes contradictory. Studies report both converging and diverging results regarding microbial diversity and specific taxonomic changes after different surgical techniques [15,16]. Moreover, few studies employ a longitudinal design or compare microbial changes across different bariatric approaches.

In this context, our study aimed to evaluate the dynamics of the gut microbiota in patients with obesity undergoing either LSG or RYGB. This analysis seeks to clarify the extent to which these surgical interventions influence the composition of the gut microbiota, thereby contributing to weight loss. Such research is essential for understanding the long-term effects of medical or surgical interventions on health, as well as for tracking temporal changes in biological parameters and overall patient outcomes. Using 16S rRNA gene sequencing and a longitudinal design, we assessed the microbiota composition and diversity at multiple time points, before and after surgery. The novelty of this study lies in the inclusion of three distinct sampling moments (preoperative, 3 months, and 6 months postoperatively) in the same patients, combined with a normal-weight control group for direct comparison, and the use of high-resolution next-generation sequencing targeting the V3–V4 regions of the 16S rRNA gene. Few previous studies have integrated these elements to capture the temporal trajectory of gut microbiota changes after both LSG and RYGB in a single protocol. Our findings provide insight into the microbial mechanisms potentially contributing to the clinical success of bariatric surgery and offer a foundation for future personalized interventions targeting the gut microbiome.

## 2. Results

In the study group, consisting of 39 patients with obesity undergoing bariatric surgery, 9 participants (23.1%) were male and 30 participants (76.9%) were female. The mean age in this group was 39.12 ± 10.4 years (range: 22–62 years), with a median of approximately 39 years. The control cohort included 27 normal-weight individuals, of whom 8 (29.6%) were male and 19 (70.4%) were female. Their mean age was 36.92 ± 9.23 years (range: 25–61 years), with a median of approximately 33 years. The age and sex distributions are important variables to consider when assessing changes in the gut microbiota following bariatric surgery, as both factors may influence the microbial composition.

In terms of BMI, the study group had a mean BMI of 42.59 ± 5.61 kg/m^2^, with values ranging from 34.45 to 57.44 kg/m^2^. The control group presented a mean BMI of 23.95 ± 3.02 kg/m^2^, with a minimum of 15.43 kg/m^2^ and a maximum of 29.83 kg/m^2^.

Throughout the follow-up period—at baseline (T0), three months (T3), and six months (T6) postoperatively—the study group exhibited a consistent downward trend in mean BMI values. At three months post-surgery (T3), the mean BMI was 36.57 ± 4.82 kg/m^2^, with values ranging from 28.72 to 43.77 kg/m^2^. At six months (T6), the mean BMI decreased further to 31.08 ± 4.49 kg/m^2^, with a minimum of 24.84 kg/m^2^ and a maximum of 40.48 kg/m^2^.

The most prevalent comorbidities associated with excess weight in the study group were metabolic dysfunction-associated fatty liver disease (92.3%), obstructive sleep apnea (76.9%), vitamin D deficiency (66.7%), hepatomegaly and dyslipidemia (64.1%), essential hypertension (41.0%), impaired glucose tolerance (23.1%), and type 2 diabetes mellitus (T2DM) (15.4%). Additionally, four patients had known hypothyroidism under treatment and two were diagnosed with gastroesophageal reflux disease (GERD). Among the 39 patients in the study group, 53.85% were on chronic medication, while 46.15% were not. The main medication classes included antihypertensives, oral antidiabetics, statins, and synthetic thyroid hormones.

The initial sequence reads had a maximum length of 150 base pairs. After successive filtering steps, sequence lengths ranged between 119 and 134 base pairs. A total of 13,384,584 sequences were generated across all 96 samples (93 patient samples, 1 duplicate, and 2 negative controls). Following quality filtering, 11,832,839 sequences remained, of which 3978 were unique. On average, 88.82% of the sequences were retained after filtering. Samples F8-1, F15-1, and F30-2 were excluded due to low DNA concentrations, as determined via spectrophotometric quantification.

Post-filtering, the number of sequences per sample ranged from 657 to 759,444. Three samples—two negative controls and sample F11-3—had fewer than 5000 reads and were excluded from the analysis, as such low counts are unlikely to represent the true microbial composition of the sample.

Filtering of the sequences of interest resulted in the retention of 3605 unique sequences, which were classified at the phylum level (Table 1).

Following the data processing workflow, a total of 3599 unique sequences were identified across all 93 samples. Among these, some sequences could not be taxonomically resolved to the species level but were assigned to the same higher taxonomic rank (e.g., two sequences unclassified at the species level but belonging to the same genus). To reduce dimensionality and complexity, sequences sharing identical taxonomic classification were aggregated at the most detailed common taxonomic level. After this clustering process, 587 unique sequences remained across the entire dataset.

**Figure 1:**
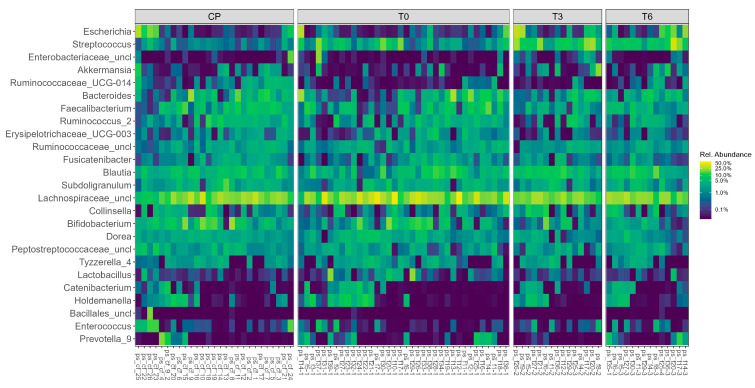
Heatmap of bacterial genus abundance in gut microbiota samples from the surgical group and control group.

The relative abundance of the most representative bacterial genera across all 93 samples is illustrated in Figure 1.

Notably, genera such as *Bacteroides*, *Akkermansia*, *Bifidobacterium*, *Faecalibacterium*, and *Prevotella* were consistently present across multiple samples, though with varying levels of abundance. Some phyla, including *Collinsella*, *Dorea*, and *Lactobacillus*, showed pronounced variability between individuals. The heatmap reveals clear inter-individual differences in the microbiota composition, which may be associated with clinical or procedural factors.

**Figure 2:**
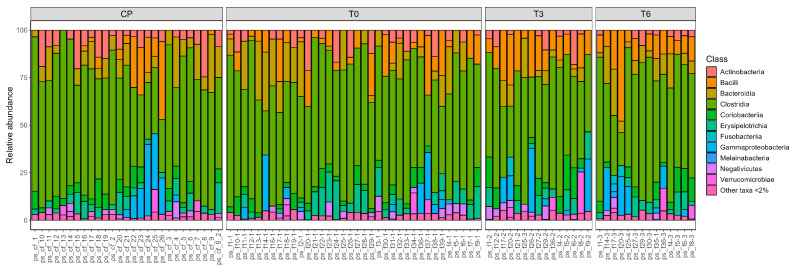
Class-level microbial composition of the analyzed samples, grouped by sampling time.

**Figure 3:**
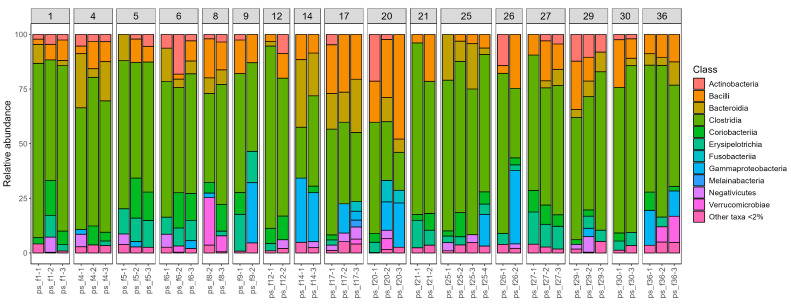
Class-level microbial composition of the analyzed samples, grouped by patient. Only samples with more than one time point were included in this representation.

In addition to a genus-level analysis, we also generated visual representations of the taxonomic composition at the class level for all samples. These were grouped according to the sampling time (Figure 2) and by individual patient (Figure 3), to better observe temporal dynamics and inter-individual variation.

**Figure 4:**
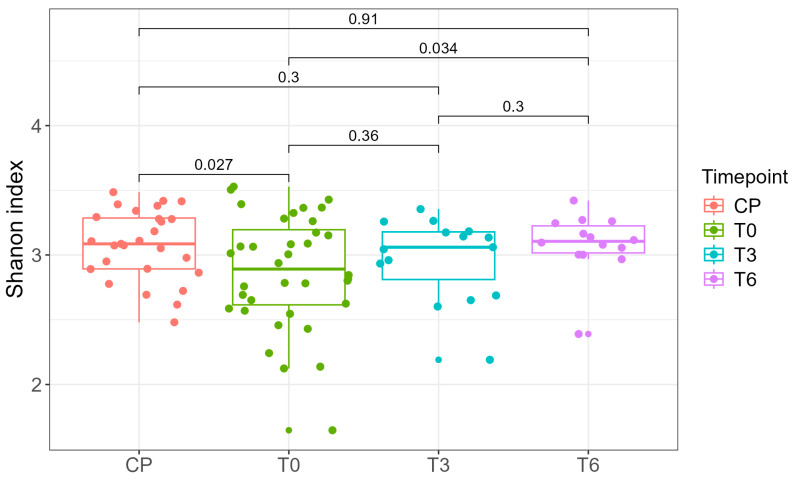
Shannon index as a measure of alpha diversity in normal-weight controls (CP) and patients at preoperative (T0), 3-month (T3), and 6-month (T6) time points. Group comparisons are indicated by brackets, with corresponding *p*-values displayed above. Statistical significance was considered for *p* < 0.05.

We calculated several alpha diversity indices, including the Shannon index and the Inverse Simpson index. Briefly, a lower Shannon index indicates reduced bacterial diversity within a sample, which makes it easier to identify samples dominated by a single or limited group of bacterial taxa. Statistically significant differences in the Shannon index were observed between the control group (normal-weight individuals) and the preoperative samples (T0) (*p* = 0.027, Student’s *t*-test), as well as between preoperative (T0) and six-month postoperative (T6) samples (*p* = 0.034, Student’s *t*-test) (Figure 4). No other statistically significant differences were identified between sample groups.

**Figure 5:**
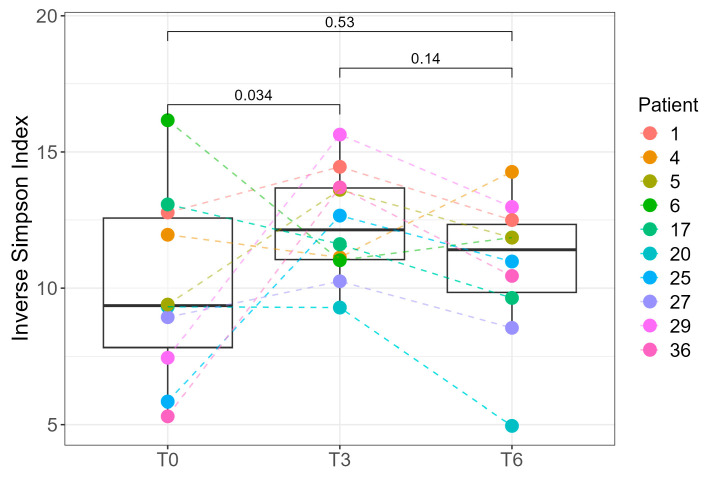
Inverse Simpson index as a measure of alpha diversity in patients at preoperative (T0), 3-month (T3), and 6-month (T6) time points. Comparisons are marked with brackets and *p*-values above. *p* < 0.05 was considered statistically significant. Samples are color-coded by patient, and dotted lines represent the temporal trend of the index for each individual.

Due to the availability of complete sampling (T0, T3, and T6) for only 10 of the 39 patients, we performed paired statistical analyses exclusively for this subset. A statistically significant difference in the Inverse Simpson index was observed between T0 and T3 (*p* = 0.034, paired Student’s *t*-test), indicating changes in intra-individual microbial diversity over time (Figure 5). At the cohort level, Shannon diversity did not differ between T0 and T3 (*p* = 0.36), likely due to inter-individual differences and the smaller number of T3 samples, which reduce power for between-group tests. In the 10 patients with complete follow-up, a paired analysis of the Inverse Simpson index detected an increase from T0 and T3 (*p* = 0.034), indicating a within-person change that the unpaired comparison may miss. Because the Inverse Simpson index up-weights dominant taxa, whereas the Shannon index is more sensitive to richness/evenness, early postoperative shifts likely involve changes among higher-abundance genera rather than broad gains in rare taxa. By 6 months, Shannon diversity was higher than at baseline in the full cohort (T6 vs. T0, *p* = 0.034; Student’s *t*-test; Figure 4), consistent with postoperative stabilization of the gut ecosystem: as diet diversifies and host physiology normalizes, dominance by a few taxa diminishes, while several commensal genera reach moderate relative abundances, increasing evenness and thereby elevating the Shannon diversity.

**Figure 6:**
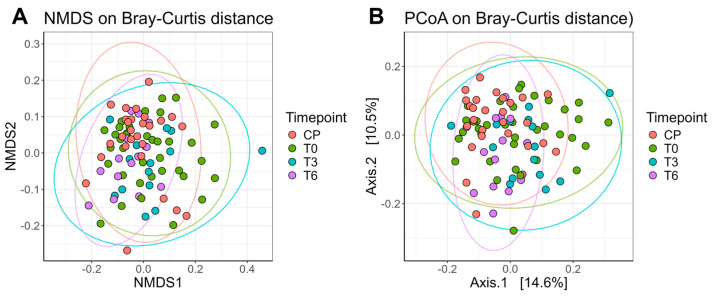
(**A**) NMDS and (**B**) PCoA plots based on Bray–Curtis dissimilarity, illustrating beta diversity among bacterial communities in the samples. Samples are color-coded by group. Ellipses represent 95% confidence intervals. Axis labels include the variance coefficient; in the PCoA plot, the percentage of explained variance for the first two dimensions is also shown.

We also calculated the Bray–Curtis dissimilarity index as a measure of beta diversity, which reflects the compositional similarity between samples. All available samples were included in the beta diversity visualizations (Figure 6). The ellipses represent, in simple terms, the “spread” of data points. Samples collected at T6 showed a narrower dispersion compared to those from T0 or T3, with centroids shifting closer to those of the control group (normal-weight individuals). Consistent with the alpha-diversity pattern, dispersion at T6 was lower than at T0 (FDR-adjusted *p* = 0.0266), with no difference vs. T3 or controls, and the ordination centroids at T6 shifted toward controls. Thus, by 6 months, we observe higher within-sample evenness (Shannon) together with reduced between-sample spread (Bray–Curtis dispersion)—a coherent signature of postoperative stabilization.

**Figure 7:**
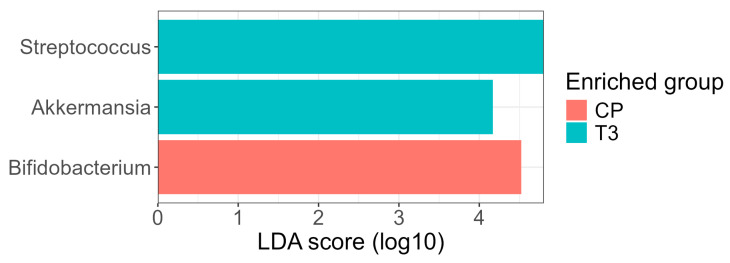
LEfSe analysis of differential bacterial genera abundance across groups. The significance threshold was set at an LDA score of 4.

Differential abundance analysis using the LEfSe method identified three genera with LDA ≥ 4.0 showing significant differences between groups (Figure 7). At the three-month postoperative time point (T3), the genera *Streptococcus* and *Akkermansia* were enriched, whereas the genus *Bifidobacterium* was enriched in the control (normal-weight) group. The increase in *Akkermansia* at T3 is noteworthy given its recognized role in maintaining mucosal integrity, modulating inflammation, and improving glucose metabolism; previous studies have reported an expansion of *Akkermansia muciniphila* following RYGB surgery [17]. *Bifidobacterium*, a commensal genus often associated with metabolic health and dietary fiber fermentation, was more abundant in the control group, consistent with its frequent reduction in individuals with obesity and its reported recovery after interventions promoting metabolic balance.

The number and identity of taxa detected were influenced by the choice of significance thresholds for the Kruskal–Wallis and Wilcoxon steps during the LEfSe analysis. The use of a more conservative *p*-value threshold (*p* < 0.01) retained Streptococcus as the sole significant taxon, while the more conventional *p* < 0.05 threshold revealed the additional differential abundance of *Akkermansia* and *Bifidobacterium*. This reflects the inherent trade-off between specificity and sensitivity in biomarker discovery: stringent thresholds prioritize robustness, whereas more permissive settings can reveal additional biologically plausible taxa that may be otherwise overlooked.

## 3. Discussion

### 3.1. General Aspects of the Study

The present study investigated the effects of bariatric surgery on the gut microbiota in obese patients, following the evolution of the microbiota at three different time points: before surgery (T0), three months (T3) and six months (T6) postoperatively. This aspect captures the dynamic evolution of the bacterial composition after surgery. This longitudinal approach helps in understanding how the microbial community changes over time and does not provide only a snapshot, which is a valuable contribution to the existing literature. On the other hand, many studies focus on postsurgical outcomes at a single time point, which may not capture the full spectrum of microbiota evolution. The inclusion of a control group of normal-weight individuals in this study was essential because it provides a necessary baseline to assess the specific impact of bariatric surgery on the gut microbiota, allows the effects of the intervention to be distinguished from natural variations in the microbiota between individuals, and helps control for confounding variables. It also increases the external validity of the study, making the results more applicable and generalizable to a broader population context. Thus, we can highlight the changes in the microbiota that are directly related to weight loss and metabolic improvements post-surgery, providing a solid scientific basis and contributing to more precise and reliable conclusions about the relationship among obesity, surgery, and the gut microbial composition. One of the distinctive features of this study was the use of next-generation sequencing (NGS) technology to analyze the composition of the gut microbiota. This new approach provides a much more detailed and deeper view of the diversity and composition of the microbiota than traditional methods. By using NGS, not only quantitative but also qualitative changes in the microbiota can be identified, including specific variations at the level of bacterial genera and species.

This methodological approach expands our understanding of the complex interactions among diet, bariatric surgery, and the gut microbiota and opens up avenues for future research. It also highlights the potential of microbiota changes as biomarkers for assessing individual responses to surgical treatments and for personalizing therapies based on each patient’s microbial profile. Furthermore, the application of NGS in this study represents a step forward towards personalizing medicine and improving obesity management and may contribute to establishing a new paradigm in the treatment of obesity and its comorbidities.

Among the main findings, the study highlighted a significant decrease in BMI in the surgical group, correlated with changes in the composition and diversity of the microbiota. These changes indicate an association between weight loss and transformations in the gut microbiota, which are consistent with postoperative physiological changes. The results also highlight an increase in bacterial diversity in samples taken 3 months postoperatively (T3), evidenced by the increase in differential abundance for bacteria in the *Actinobacteria* class, suggesting possible normalization of the microbiota composition post-intervention. The results also indicate that changes in bacterial communities could be a positive side effect of surgery, possibly contributing to decreased chronic inflammation and improved metabolism.

While samples taken 6 months postoperatively (T6) revealed a continuation of the trend of normalization of the microbiota composition, compared to normal-weight patients, no significant differences were found between T3 and T6. The result suggests that the most notable changes in the intestinal microbiota occur in the first three months post-surgery.

An important aspect is that the impossibility of classification down to the species level is related to the overlap of the sequence based on the amplified region within the NGS flow in several species within the same genus. For example, if we were to search for the first sequence in the 16S rRNA database (Bacteria and Archaea type strains), we would observe 100% matches at the same level between the following species: *Blautia intestinalis* strain 27–44, *Blautia luti* DSM 14534, and *Blautia wexlerae* DSM 19850, but also with *Blautia obeum* ATCC 29174.

Of course, assumptions can be made about the actual species-level classification, depending on the probability of encountering the species in a geographical context. For example, *Blautia luti* was isolated in 1999 from a patient in Germany, *Blautia wexlerae* was isolated in 2007 from a patient in the USA, and *Blautia intestinalis* strain 27–44 was isolated in 2021 from a patient in China [18]. On the other hand, *Blautia obeum*, with which it is 99.25% identical (i.e., differing by a single nitrogen base), has been isolated from patients worldwide, including Europe, and is among the most prevalent bacteria in fecal samples. Therefore, there may be a small sequencing error in the experiment, which makes the match between this sequence and the consensus sequence from *Blautia obeum* not 100%, but in fact, it comes from this species. However, the incomplete sequence match for all nitrogenous bases makes it impossible to classify according to the classification algorithm down to the species level.

In addition to observations related to the dynamics of the intestinal microbiota composition, the study also monitored the prevalence of pathologies associated with excess weight. As demonstrated in previous investigations, the main comorbidities in the study group, in order of frequency, were represented by fatty liver disease associated with metabolic dysfunction, sleep apnea syndrome, vitamin D deficiency, lipid metabolism disorders, arterial hypertension, and low arterial glucose.

These results are consistent with previous studies that have reported changes in the gut microbiota after bariatric surgery and suggest that changes in the microbiome may play a role in the mechanisms by which surgery leads to weight loss and improved health. However, the need for long-term follow-up to investigate the durability of these changes and the impact on long-term health remains evident. Finally, the findings provide promising insight into the potential benefits of surgical approaches for the treatment of obesity, highlighting a new area of research related to the human microbiome and metabolic health.

### 3.2. Aspects Related to Bacterial Diversity

Alpha diversity is a measure of biodiversity within a single sample or habitat, and it captures the variety and abundance of species present. This concept is central to research on the complexity and ecological variation of individual samples, focusing primarily on the number of distinct species within them. The quantification of alpha diversity includes various indices and notably emphasizes the prevalence of different species. One of the fundamental metrics of alpha diversity is known as taxonomic diversity. This measure calculates the number of unique microbial entities identified in a sample at a given taxonomic level, such as the species. Terms such as “observed species” frequently appear in research, signifying the diversity observed at various taxonomic scales [19].

Another essential index is the Shannon Index, also known as the Shannon diversity index or Shannon entropy. It provides an assessment of species abundance and evenness, giving greater weight to the aspect of abundance. The principle underlying this index is that the diversity of a sample increases with the number of distinct species in its distribution. Essentially, it reflects the uncertainty in predicting the identity of the next species observed in a sample. Similarly, the Simpson Index (or Gini-Simpson), with various derived formulas, is widely applied. Conceptually, it is similar to the Shannon index but is based on the probability that two microbial entities selected at random from a sample represent certain different species [20].

Phylogenetic diversity adds another dimension to the assessment of alpha diversity. Unlike other metrics that focus solely on the number and abundance of species, it assesses the genetic lineage diversity among observed species and calculates the total length of all phylogenetic branches connecting species in a sample [19]. This measure provides a broader understanding of diversity, emphasizing genetic variation beyond the simple number of species. In research contexts, examining alpha diversity helps to compare biodiversity between different groups or monitor changes within a group over time. It serves as a fundamental building block for understanding the ecological and genetic composition of environments or communities [21].

Beta diversity is a term used in ecology to describe the comparative degree of variation or differences between samples or habitats. It is frequently used in microbiome studies to discern significant variations between distinct groups, such as experimental and control groups [20].

The Bray–Curtis Dissimilarity Index plays an important role in assessing similarities and differences in microbial abundance between two different samples. The Bray–Curtis Index scale ranges from 0 to 1, where a value of zero denotes identical microbial numbers and abundances in both samples, while a value of one indicates complete disparity, with no shared microbial species [22]. This indicator is preferred due to its limited range, facilitating a straightforward and simplified interpretation of sample similarity. However, the fixed scale can exaggerate or minimize differences, especially concerning the number of bacteria in the samples. For example, small variations in abundance in samples with fewer microbes can significantly influence the Bray–Curtis dissimilarity score, while in samples with a high number of species, similar changes in abundance might have a smaller impact. Therefore, it is recommended to also consider alpha diversity when assessing differences between samples [20].

In our study, microbial diversity, measured based on the Shannon and Inverse Simpson indices, highlighted significant differences between pre- and postoperative times, indicating a dynamic of bacterial diversity over time. In terms of the Shannon Index, statistically significant differences were observed between the CP and T0 groups and the T0 and T6 groups, respectively. The smallest differences in terms of diversity (similarities) were observed between the CP and T6 groups. Also, between the T0 and T3 groups, as well as T3 and T6, the differences were minor but constant from one time point to another. The results reflected an increase in microbial diversity over time and a trend toward “normalization” of the intestinal microbiota in obese patients who underwent bariatric surgery, compared to the control group of normal-weight patients.

In terms of beta diversity, multivariate analysis revealed a significant difference in the microbiota composition, influenced by the time of sampling. It was also found that the study group and the normal weight control group differed significantly at all three sampling times, suggesting a significant influence of bariatric surgery on the composition of the intestinal microbiota. From the analysis of the Bray–Curtis dissimilarity index and the graphical representation of distances using the NMDS and PCoA techniques, it resulted that the data are less “scattered” at time T6 (agglutination tendency). From a compositional point of view, the patients are more similar in terms of microbiota compared to times T0 and T3, where the data are more dispersed. Also, the center is closer to those of the control group (norm-weight patients), a result that again captures the trend of normalization of the composition of the intestinal microbiota, both qualitatively (diversity) and quantitatively. These changes reflect the potential mechanisms by which bariatric surgery contributes to improving the health status and reducing obesity-related comorbidities.

### 3.3. Alignment with Previous Research

One of the landmark studies in this field, conducted by Jeffrey Gordon and his collaborators, has profound implications for biomedical research and understanding the link between microbiota and obesity. The study involved the transfer of gut microbiota from monozygotic human twins discordant for obesity to laboratory mice raised in a sterile environment. This research demonstrates that the gut microbiota plays a significant role in regulating metabolism and can directly influence the predisposition to obesity, in addition to modifying eating behavior. This highlights that future therapeutic approaches could include manipulating the composition of the gut microbiota through methods such as fecal microbiota transplantation, the administration of probiotics and prebiotics, or specific dietary adjustments to combat obesity and associated metabolic disorders [23].

Furthermore, the results of the study point to the possibility of personalizing obesity treatments, taking into account the unique composition of each individual’s gut microbiota. This suggests that future interventions may be more effective if they are tailored to the specific microbial profile of each patient, marking an evolution towards more personalized and less invasive approaches. Finally, the use of mice raised in sterile, non-colonized conditions highlights the value of animal models in exploring the relationships between the microbiota and the host organism, facilitating the development and testing of new therapeutic interventions. Therefore, the study marked a turning point in microbiome research and provided new insights into the potential of the gut microbiota as a therapeutic target in obesity and other metabolic diseases. A preliminary study conducted exclusively in laboratory animals, led by Turnbaugh and colleagues in 2006, is one of the first to highlight the direct role of the gut microbiota in regulating metabolism and contributing to the development of excess weight. This research demonstrates that the transplantation of gut microbiota from obese mice to germ-free mice (without their microbiota) leads to a significant increase in the weight of the recipients, even when they were fed a standard diet. This result suggests that the gut microbiota can influence the efficiency with which energy is extracted from food and stored [24].

The implications of this study are vast and varied. First, it has changed our understanding of the factors that contribute to obesity, highlighting a significant role for the gut microbiota, which until then was considered less relevant to host metabolism. This has opened the way for new research directions that examine how different factors, such as diet, genetics, and the environment, can influence the composition and function of the microbiota and, by extension, the risk of obesity and other metabolic disorders. The research has contributed to the development of a new field of study that combines microbiology, nutrition, and endocrinology to better understand the complexity of biological systems. It has encouraged researchers to look beyond the simple relationship between caloric intake and body weight and to consider a much more dynamic and interconnected system that includes the gut microbiota.

A recently published study explores the effect of human gut microbiota transplantation after bariatric surgery on blood glucose levels and intestinal changes in rodents [25]. The study included obese female patients with T2DM who underwent either biliopancreatic diversion (BPD-DS) or LSG. Fecal samples collected before and after surgery were used to colonize the digestive tracts of germ-free mice to examine the impact on glycemic control. The main results were improved glucose tolerance in germ-free mice colonized with human microbiota from patients 12 months after surgery, with no changes in food intake, fat mass, or insulin resistance, secretion, or clearance. Notably, these mice demonstrated changes in the gut morphology, such as decreased villi height/width and crypt depth in the distal jejunum, along with reduced intestinal glucose absorption. The improvements in blood glucose control mediated by the microbiota were linked to inhibition of the sodium-glucose cotransporter (SGLT)1, indicating that these benefits depend on reduced intestinal glucose absorption. The study also noted changes in bacterial populations, with an increase in *Parabacteroides* and a decrease in *Blautia* populations, associated with improved blood glucose control in mice colonized with the post-operative microbiota. The study concludes that exposure to the human gut microbiota after bariatric surgery, either restrictive or malabsorptive, improves glycemic control in rodents. This suggests that the post-surgical microbiota independently influences blood glucose levels by altering the gut morphology and reducing Sglt1-mediated glucose absorption, regardless of changes in BMI, insulin, or insulin resistance [25].

Another literature review focuses on the complexity of the interaction between the gut microbiota and bariatric surgery and how these procedures induce anatomical and physiological changes that affect the microbial ecosystem. The compositional changes are related to improved carbohydrate metabolism, changes in eating behavior, body weight reduction, improved gastrointestinal motility, and an improved nutritional status. In addition, microbial metabolites, including short-chain fatty acids and secondary bile acids, betaine and choline, may have a beneficial synergistic role in human metabolism and in promoting weight loss [26]. A significant decrease in acetate, butyrate, and propionate concentrations was also observed [27].

The observed studies indicate the remission of T2DM after surgery in 80% of patients at 6 months postoperatively and highlight an association between weight loss and a reduction in insulin resistance. In patients who experienced remission, a significant increase in populations of *Roseburia intestinalis* from the phylum *Firmicutes* was also observed, regardless of the type of surgical procedure performed, associated with beneficial effects on insulin sensitivity. These results support the hypothesis suggesting that changes in the gut microbiota after bariatric surgery may be linked to T2DM remission [28].

Several studies on the intestinal flora of obese patients document a higher relative abundance of *Firmicutes* phylum representatives compared to *Bacteroidetes*, but also a lower microbial diversity [29]. However, changes in the proportion of the *Firmicutes* phylum after this type of surgery were heterogeneous, and most of the studies investigated report contradictory results regarding the *Firmicutes*/*Bacteroidetes* (F/B) ratio [30]. As demonstrated by the results of our study, in percentage terms, representatives of the *Firmicutes* phylum dominate the landscape of microbial diversity. The F/B ratio was 7.7 at time T0, 12.6 at time T3, and 7.3 at time T6 vs. 9.3 in the group of normal-weight patients. Although significantly altered at T0 compared to T6, a particularly important role is played by the restoration of both qualitative and quantitative microbial diversity by increasing the abundance of representatives of the phyla *Actinobacteria*, *Proteobacteria*, and *Verrucomicrobia*. The increase in the abundance of *Proteobacteria* has also been observed in other studies, at 6 months postoperatively, both after RYGB and LSG, an effect that is most likely due to better exposure of the intestinal mucosa to oxygen and changes in pH in the digestive tract [31]. Some studies on murine models associate a higher abundance of this phylum with improved insulin sensitivity and suggest a beneficial role in glucose metabolism.

Among bariatric surgeries, it has been observed that RYGB significantly alters the composition of the gastrointestinal microbiota compared to LSG. Although both procedures recommend similar dietary recommendations and manage post-surgical eating habits, supporting weight loss and helping to control T2DM, RYGB tends to induce important functions in the microbiota, influencing the multiplication of factors such as intestinal motility, bile acid secretion and metabolism, and levels of various incretins [32].

Although both RYGB and LSG are associated with significant changes in the microbiota composition, the results remain controversial and may vary depending on the postoperative period studied. It is unclear whether these changes are permanent or whether they are a direct result of the surgical procedures themselves or of dietary and lifestyle changes that occur after surgery. Caution is also required in assessing the magnitude of the influence of microbiota changes as a contributing factor in promoting weight loss and metabolic improvement postoperatively [33]. Although our present analysis combined LSG and RYGB to preserve statistical power, we acknowledge that the procedures may influence the microbiota through partially distinct mechanisms (e.g., bile acid dynamics and nutrient flow after RYGB vs. gastric restriction and altered ghrelin after LSG). A larger cohort is planned to enable reliable subgroup comparisons and highlight surgery-specific microbiota effects, minimizing the risk of false-negative findings in procedure-stratified analyses.

A bacterial species with an important role in regulating the immune response and the balance of metabolic activity is *Akkermansia muciniphila*, a representative of the phylum *Verrucomicrobia*. It is recognized for its role in supporting weight loss and improving T2DM remission rates after bariatric surgery. This bacterium showed relatively high abundance in four out of 17 clinical trials involving RYGB and in three out of nine studies associated with LSG [26].

### 3.4. Study Limitations, Challenges, and Strengths

This study has significant strengths, including its prospective longitudinal approach that facilitates the observation of the long-term evolution of the microbiota. The inclusion of a normal-weight control group adds important comparative value, while obtaining informed consent and ethical approvals emphasizes compliance with ethical principles, strengthening confidence in the results.

Inter-individual variability in the gut microbiota composition, together with potential inaccuracies inherent to the self-collection of biological samples, represent methodological constraints. The single-center design, although ensuring consistency in patient selection, surgical techniques, and sample processing, may limit the extrapolation of the results to other populations or clinical settings. Demographic, dietary, and lifestyle particularities specific to our region could also influence the gut microbiota composition. In addition, external factors such as postoperative dietary habits, lifestyle modifications, and levels of physical activity were not systematically monitored or controlled, which may have contributed to the observed microbial changes. Finally, the study design does not allow us to establish whether these alterations are a cause or a consequence of the postoperative evolution; further mechanistic and interventional studies are required to clarify this relationship.

There are also potential sources of bias, including selection bias, which can occur when the characteristics of participants who choose to remain in the study differ from those who withdraw. Measurement and reporting bias can also influence the integrity of the data collected. To counteract these limitations and biases, the application of rigorous study protocols, standardization of sample collection methods, and the use of advanced statistical models are essential. Effective communication with participants, providing clarification and explaining the importance of the study and participation until the end of the follow-up period, can improve the retention and accuracy of reported data, contributing to more reliable results and a deeper understanding of the impact of bariatric surgery on the gut microbiota.

## 4. Materials and Methods

### 4.1. Study Inclusion, Sample Collection, and Storage

This was a prospective, observational, single-center study designed to investigate changes in the gut microbiota among patients with obesity undergoing bariatric surgery, either LSG or RYGB. The procedures were performed at the “Sf. Spiridon” Emergency Clinical Hospital in Iași, within the Bariatric Surgery Center of the Third Surgical Clinic. Study participants were enrolled following their written informed consent, in accordance with the Declaration of Helsinki. The study protocol was approved by the Ethics Committee of the “Grigore T. Popa” University of Medicine and Pharmacy and the Institutional Ethics Committee of “Sf. Spiridon” Emergency Clinical Hospital (approval no. 137/25.01.2022 and no. 34/17.03.2022, respectively, for studies involving human subjects).

Inclusion criteria consisted of eligibility for bariatric surgery based on BMI values, the presence of obesity-related comorbidities, the recommendation of a multidisciplinary evaluation team, and signed informed consent. Exclusion criteria included pre-existing gastrointestinal disorders, recent use of antibiotics, psychiatric disorders, neoplasia, and refusal to participate in the study.

The methodology involved the collection of stool samples (approximately 1 cm^3^) in sterile containers at three time points: one day prior to surgery (T0), three months postoperatively (T3), and six months postoperatively (T6). After collection, samples were transported to the molecular biology laboratory. If processed immediately, they were stored at 4 °C; otherwise, they were frozen at −80 °C until DNA extraction.

Following the application of predefined exclusion criteria, 66 participants were deemed eligible and were randomized into two groups: 39 obese patients scheduled for bariatric surgery (intervention group) and 27 age-matched normal weight individuals (control group).

The minimum sample size was determined based on information from the literature, which indicates that 1% of clinically eligible patients undergo surgical treatment for obesity [34]. Establishing the optimal sample size required ensuring a minimum volume to achieve adequate representativeness of the patient population. To meet this prerequisite, a 95% confidence interval was established. Consequently, the following equation was used:

$$n\geq {\left(Z_{\left(1-\frac{\alpha }{2}\right)}\right)}^{2}\times \frac{p\left(1-p\right)}{d^{2}}$$

with *Z* = 1.96 for a 95% confidence interval and a “*d*” value corresponding to an estimation error of 5%. For a maximum assumed error of 5%, the minimum sample size was calculated to be 16 cases (*n* ≥ 15.21). Thus, in this study, from a total of 66 patients, the application of inclusion and exclusion criteria resulted in a study group comprising 39 cases.

### 4.2. Experimental Design for Illumina 16S rRNA Gene Sequencing

The experimental design for Illumina 16S rRNA gene sequencing includes sample collection, DNA extraction, library preparation, and sequencing parameters. Special attention should be paid to primer design, as the choice of variable regions influences the resolution of taxonomic classification. After obtaining the raw sequencing data, a comprehensive bioinformatics analysis is of paramount importance. The detailed protocol for DNA extraction and sequencing was previously described [35,36].

The 16S V3–V4 region is a frequently targeted region for sequencing of the bacterial 16S rRNA gene. This region covers a variable area between the third and fourth hypervariable regions of the gene and is often used in bacterial diversity studies. When sequencing the 16S V3–V4 region with Illumina technology, the general protocol involves the following steps:

- Sample collection and extraction of bacterial genetic material;
- Library preparation by amplifying the target region (16S V3–V4) using PCR primers designed for this purpose and attaching sequencing adapters to the amplicons to create a DNA library;
- Quantification of the DNA library concentration to ensure that we have enough material for sequencing;
- Normalization of the DNA library concentration to ensure equal representation of the samples;
- Generation of DNA clusters on the Illumina flow-cell, with each cluster representing a group of identical DNA fragments;
- Sequencing the library using the Illumina sequencing platform with 150-base-pair reads on both ends;
- Performing bioinformatic analysis on the raw sequencing data;
- Trimming and filtering low-quality reads;
- Assembling the reads on both ends into contigs;
- Assigning taxonomy to sequences (assigning each sequence to a taxonomic group);
- Performing diversity and statistical analyses.

In order to optimize the breakdown of both Gram-negative and Gram-positive bacteria, incubation times were tested. Enzyme concentrations were also tested for three different enzymes, with the subsequent addition of lysostaphin (Sigma-Aldrich Co., St. Louis, MO, USA), lysozyme, and proteinase K (Thermo Fisher Scientific, Waltham, MA, USA).

### 4.3. Negative Controls

Several negative DNA extraction controls (4 per run) without fecal matter, but otherwise handled in the same way as the samples, were performed throughout the process, including polymerase chain reaction (PCR) amplification (absence of visible bands) to identify possible contaminations.

### 4.4. Quality Assessment and Quantification of Extracted DNA

The concentration of extracted DNA (absorbance at 260 nm) and its purity (absorbance ratios 260/230 and 260/280) were determined spectrophotometrically using a NanoDrop (Thermo Fisher Scientific, Waltham, Massachusetts, USA). Ratios of 1.8 to 2.0 suggest the absence of protein contamination.

To determine the bacterial composition of each sample, we generated a 16S metagenomic sequencing library, according to Illumina instructions. Briefly, we amplified the V3-V4 region of the 16S rRNA gene with a PCR primer using the following specific primer sequences with adapters at the F ends: 5′—TCGT CGGC AGCG TCAG ATGT GTAT AAGA GACA GCCT ACGG GNGG CWGC AG—3′ and R: 5′—GTCT CGTG GGCT CGGA GATG TGTA TAAG AGAC AGGA CTAC HVGG GTAT CTAA TCC—3′, which generated an amplicon of approximately ~460 base pairs.

### 4.5. Data Analysis

The demultiplexed sequences were processed using the dada2 v1.22 package, implemented in R. Briefly, primers were trimmed from the sequences, after which sequences containing ambiguous bases (N) were removed. We decided not to further trim the sequences, as the quality profiles indicate a high sequencing quality. We removed sequences containing bases with a quality score of less than 2, and a maximum of 2 sequencing errors per cent of nucleotides was considered (the maxEE parameter was set to 2); then, we removed chimeric reads (amplification artefacts often encountered in 16S rRNA sequencing, caused by incomplete extensions during PCR cycles).

Taxonomic classification of the sequences after filtering was performed using the DADA2 implementation of the Ribosomal Database Project (RDP) naive Bayesian classifier, trained on the V3–V4 region of the sequences from the SILVA database (version 132), down to the species level (where possible, by exact matching). Amplicon sequence variants (ASVs) classified as Archaea, eukaryotes, mitochondria, or chloroplasts, as well as those not classified at the phylum level, were eliminated. Samples that, after filtering, remained with a sequence count of less than 5000 were eliminated from the study. Phyloseq objects containing ASV tables, taxonomic classifications, and metadata were built with the phyloseq v 1.38 package.

Heatmaps were generated using the phyloseq package and display relative abundances (percent) on a log-transformed color scale to enhance the visibility of low-abundance taxa in a right-skewed distribution.

Nonphylogenetic alpha-diversity indices were calculated with the phyloseq v1.38 package. The Bray–Curtis dissimilarity matrix was constructed using phyloseq, starting from data transformed via variance-stabilizing transformation. Two-dimensional reduction methods were used: NMDS (non-metric multidimensional scaling) and PCoA (principal coordinates analysis).

The distance to the centroid of the group variances was calculated via multivariate analysis of homogeneity of group variances using the vegan v2.6-4 package. PERMANOVA was performed with the same package, and the paired PERMANOVA analysis was performed using the RVAideMemoire v0.9-81-2 package with the *p*-index correction method using the FDR method of Benjamini and Hochberg. To verify that between-group differences were not driven by unequal dispersion, we tested the homogeneity of multivariate dispersions and found similar within-group variance across groups.

Differential abundance analysis was performed using the LEfSe (Linear discriminant analysis Effect Size) method, using the microbiomeMarker v1.0.2 package under the permissive multi-group strategy with LDA = 4 and *p* < 0.01 or *p* < 0.05; the stringent all-against-all setting yielded no features due to a lack of consistent T3 differences vs. all comparators.

## 5. Conclusions

The present study addresses the impact of bariatric surgeries, such as RYGB and LSG, on the intestinal microbiota in obese patients, highlighting its dynamics in the postoperative period. Following the analysis, significant changes in the composition of the microbiota were found, characterized by a progressive decrease in the abundance of the phylum *Firmicutes* and an increase in the abundance of the phyla *Bacteroidetes*, *Actinobacteria*, *Proteobacteria*, and *Verrucomicrobia*, together with an increase in microbial diversity in biological samples taken after both types of surgeries. These changes reflect a potentially important role of the microbiota in the mechanisms of weight loss and improvement of the main metabolic imbalances.

The results of the study indicate variation in the *Firmicutes*/*Bacteroidetes* ratio, without a clear consistency between the different postoperative times or types of surgeries, suggesting that the specific effects of bariatric procedures on the microbiota may vary significantly from one patient to another.

Microbial diversity highlighted significant differences between pre- and postoperative times, indicating a dynamic of bacterial diversity over time. The statistically significant differences were observed between the CP and T0 groups and the T0 and T6 groups, respectively. The smallest differences in terms of diversity (similarities) were observed between the CP and T6 groups. Also, between the T0 and T3 groups, as well as T3 and T6, the differences are minor but constant from one moment to another. The results reflected the increase in microbial diversity over time and the tendency for “normalization” of the intestinal microbiota in obese patients who underwent bariatric surgery, compared to the control group of normal-weight patients.

In terms of the compositional similarity between samples, multivariate analysis revealed a significant difference in the microbiota composition, influenced by the time of sampling. The data are less “scattered” at time T6 (agglutination tendency), so compositionally, the patients are more similar in terms of the microbiota compared to times T0 and T3, where the data are more dispersed. Also, the center is closer to those of the control group (norm-weight patients), a result that again captures the tendency of normalization of the composition of the intestinal microbiota, both qualitatively (diversity) and quantitatively.

The data available are diverse and influenced by both the type of surgical procedure and the postoperative period considered. This variability underscores the need for cautious interpretation and further studies to better understand the long-term effects of bariatric surgery on the gut microbiota.

In conclusion, our research highlights the complex impact of surgical treatment on the intestinal microbiota and its potential implications for metabolic health and obesity management. The findings emphasize the importance of an integrated and personalized approach to obesity treatment that considers the interaction among diet, microbiota, and surgical intervention. These insights contribute to a deeper understanding of the potential benefits of surgical strategies in this context.

## Figures and Tables

**Table 1:** Unique sequences classified at the phylum level for the entire dataset.

**Actinobacteria**	**Bacteroidetes**	**Campylobacterota**	**Chloroflexi**	**Cyanobacteria**
394	680	3	1	20
**Firmicutes**	**Fusobacteria**	**Lentisphaerae**	**Patescibacteria**	**Proteobacteria**
2177	28	7	13	216
**Synergistetes**	**Tenericutes**	**Verrucomicrobia**	**Planctomycetes**	**Elusimicrobia**
8	45	11	1	1

## Data Availability

The data presented in this study are available on request from the corresponding author.
